# Expression of Bmi-1, P16, and CD44v6 in Uterine Cervical Carcinoma and Its Clinical Significance

**DOI:** 10.3969/j.issn.2095-3941.2012.01.009

**Published:** 2012-03

**Authors:** Mei-ying Weng, Lin Li, Shu-ying Feng, Shun-jia Hong

**Affiliations:** Department of Gynecology and Obstetrics, Sun Yat-sen Memorial Hospital of Zhongshan University, Guangzhou 510120, China

**Keywords:** Bmi-1 protein, P16 protein, CD44v6, uterine cervical carcinoma, immunohistochemistry

## Abstract

**Objective:**

Bmi-1, a putative proto-oncogene, is a core member of the polycomb gene family, which is expressed in many human tumors. The p16 protein negatively regulated cell proliferation, whereas CD44v6 is associated with proliferation as an important protein. Additionally, CD44v6 is an important nuclear antigen closely correlated to tumor metastasis. The present study aims to investigate the expression and significance of Bmi-1, p16, and CD44v6 in uterine cervical carcinoma (UCC).

**Methods:**

A total of 62 UCC, 30 cervical neoplasic, and 20 normal cervical mucosal tissues were used in the current study. The expression of Bmi-1, p16, and CD44v6 in these tissues was determined using immunohistochemical assay. The relationships among the expression of these indices, the clinicopathologic features of UCC, and the survival rate of UCC patients were also discussed. The correlation between Bmi-1 protein expression and p16 or CD44v6 protein in UCC was analyzed.

**Results:**

The expression of Bmi-1, p16, and CD44v6 was significantly high in cervical carcinoma compared with that in the cervical neoplasia and normal colorectal mucosa (*P*<0.05). The over-expression of Bmi-1 protein in UCC was apparently related to the distant metastasis (*P*<0.01) and the tumor, nodes and metastasis-classification, i.e. the TNM staging, World Health Organization (*P*<0.05). Nevertheless, the positive expression of p16 protein in UCC was not significantly associated with the clinicopathologic features (*P*>0.05). The Kaplan–Meier survival analysis showed that the over-expression of Bmi-1 significantly decreased the survival rate of UCC patients (*P*<0.05). A strong correlation indicated that there was statistical significance between the expression of Bmi-1 and CD44V6 proteins in UCC (*r*=0.419, *P*=0.001).

**Conclusions:**

The over-expression of Bmi-1 and CD44v6 protein closely correlate to the tumorigenesis, metastasis, and prognosis of UCC. Bmi-1 and CD44v6 may be used to predict the prognosis of cervical carcinoma. Bmi-1 may indirectly regulate the expression of CD44v6 in UCC patients. The positive expression of p16 protein is possibly associated with the tumorigenesis, but not with the metastasis or prognosis of UCC.

## Introduction

Uterine cervical carcinoma (UCC) is one of the most common malignant tumors in the female reproductive system. Over the past few years, the incidence of UCC has increased, and its morbidity rate has ranked second in female cancer-related deaths. The B-cell-specific moloney leukemia virus insert site 1 (Bmi-1) gene is the transcription inhibitor of the polycomb gene family members. Bmi-1 is a nucleoprotein of extensive expression that regulates the genetic transcription of HOX^[^^1^^]^ and plays key roles in cell proliferation, modulation of the self-renewal of stem cells and of oncogenesis, as well as tumor progression. As a proto-oncogene, Bmi-1 has first been found in mouse lymphoma caused by a retrovirus ^[^^2^^]^. Over the past years, several studies have confirmed that Bmi-1 is over-expressed in all tumor cells and tissues of lung cancer, colorectal cancer, breast cancer, nasopharyngeal cancer, oral carcinoma, cutaneous carcinoma, and gastric cancer ^[^^3^^–^^8^^]^. The p16 gene is an anti-oncogene closely correlated with the incidence and progress of several tumors. Given that the p16 gene is abnormal, it loses the negative regulation of cell growth, allowing phase-G_1_ cells to enter rapidly the S-phase. The excessive cell proliferation results in oncogenesis and tumor progression ^[^^9^^]^. CD44V6 is a variant CD44 molecule that mainly mediates the adhesive attraction between cells and between extracellular matrices. CD44V6 is related to the adhesive capacity of tumor cells on circumambient matrices, and its over-expression enables tumor cells to acquire the potency of metastasis ^[^^10^^]^. In the current study, the expression level of Bmi-1, p16, and CD44v6 proteins in cervical cancer was detected using immunohistochemical staining. The relationship between the expression and clinicopathologic features of UCC, as well as the survival rate of UCC patients was statistically analyzed.

## Materials and Methods

### Clinical data

Data of 62 UCC paraffin specimens, 30 cervical neoplasia (adenoma), and 20 normal cervical mucosa stored in the Pathology Department of the Sun Yat-sen Memorial Hospital of Zhongshan University, Guangzhou, China from January 2003 to January 2007 were used in the current study. All specimens were re-reviewed for definite pathological diagnosis. The 62 UCC patients were treated in the Department of Gynecology of the hospital. They had complete clinical data and at least three years worth of follow-up information. The patients did not undergo chemotherapy or radiotherapy before surgery. The ages of the patients in the 62 cases ranged from 24 to 78 years, with a median of 52. The tumor clinical staging was conducted based on the 2002 TNM Classification for malignant tumors, Union for International Cancer Control (UICC). The UCC patients were individually followed up mainly via outpatient visits and calls. All follow-up data were available for all patients. The shortest follow-up time was 13 months, and the longest was 42 months. The results of the follow-up revealed that 39 patients were still alive and 23 had died.

### Reagents and methods

Paraffin blocks were obtained for a serial section, with a thickness of 4 µm. Dimethylbenzene was used to dewax the specimens, and gradient ethanol was used for hydration. A 3% hydrogen peroxide solution was utilized to interrupt the endogenous peroxydase. Phosphate-buffered saline (PBS) (pH 7.2 to 7.4) was used to wash the sections. An EDTA (pH 8.0) antigen repair liquid was used for the high-pressure reparation of the antigen, and for cooling. Mouse-antihuman Bmi-1 monoclonal antibody (MCAB) (1:150 dilution; Upstate, USA) and mouse-antihuman p16 MCAB (1:50 dilution; Shanghai Long Island Biotech Co., Ltd) were added to the cell antigen. The cells were incubated in a water bath incubator at 37°C for 1 h and rinsed with PBS. EnVisionTM second antibody was then added, and further incubation was conducted at 37°C for 30 min. PBS was used for rinsing, and fresh diaminobenzidine solution was used for coloration. The reaction time was controlled by microscopy. Tap water was used to terminate the coloration. The Mayer hematoxylin technique was used for the pale after-staining of cell nuclei, and gradient ethanol was used for dehydration. Neural gum was used for mounting, and microscopic observation was performed. In the current experiment, the positive and negative controls were set up, and the known positive sections were used as the positive control. PBS was used as the negative control to replace the two first antibodies.

### Assessment standards of the results

The positive expression of Bmi-1 protein was mainly represented as pale-brown or dark brown particles in the cell nuclei. At times, a few light-brown ones also appear in the cytoplasm. Four different fields of vision were randomly taken via a high-power lens. The number of total cellular score and nucleus-positive cells were counted, and scoring was conducted based on the percentage of positive cells, as follows: 1 score for the positive cell rate ≤10%, 2 for the positive cell rate >10% and ≤50%, 3 for the positive cell rate >50% and ≤75%, and 4 for the positive cell rate >75%. The scoring was also conducted based on a strong or weak degree of staining, as follows: 1 for negative staining, 2 for weak staining, 3 for medium staining, and 4 for strong staining. The results were then decided by the cross product (CP) of the two scoring methods: CP≤4 was (–), >4 and also ≤8 was (+), >8 and ≤12 was (++), and >12 was (+++). The groups with (++) and (+++) were classified as the over-expression group, and those with (–) and (+) were the under-expression group. The CD44v6 protein expression was as follows: *i*) positive CD44v6 expression: positive coloration is represented by the light-brown particles, with the coloration mainly on the cell membrane. However, coloration in the cytoplasm was seen in a few cells; and *ii*) negative CD44v6 expression: the cell membrane in the sections was not chromogenic, or the number of the chromogenic cells accounted for less than 10% of the total mucous cells.

A successive observation of 10 high-power fields indicated that there were 100 tumor cells in each field. The scoring of the percentage in the positive cell count of the cell membrane was employed. The method of positive cell scoring was as follows: the number of positive cells <10% was (+), 10% to 50% was (++), and >50% was (+++). Thus, the cells with (+++) were classified as the over-expression group, and those with (+) and (++) were the under-expression group. The results of P16 protein expression were as follows: the positive expression of p16 protein was mainly the pale-brown particles in the nucleus and cytoplasm. The hyperchromatic nuclei ranked first, accompanied by pale-brown or dark-brown particles of various sizes in the cytoplasm. A successive observation of the 10 high-power fields (× 40) indicated that there were 100 tumor cells in each field, or of total of 1,000 cells. The positive cell counting of ≥10% showed a positive p16 expression, and that of <10% or uncolored cells indcated a negative p16 expression. The above results were obtained by two pathologists who conducted double-blind independent reviews of the sections.

### Statistical analysis

All statistical analyses were conducted using SPSS13.0. The differences among numeration data were analysed using the Chi-square statistical analysis. Multiple interactions were determined via logistic regression. A Kaplan-Meier survival curve was analysed via the log-rank test, and the correlation between the two indices was analysed using the Spearman correlation analysis. *P*<0.05 indicated statistical significance.

## Results

### Expression of Bmi-1, P16, and CD44v6 protein in UCC

**Figure 1:**
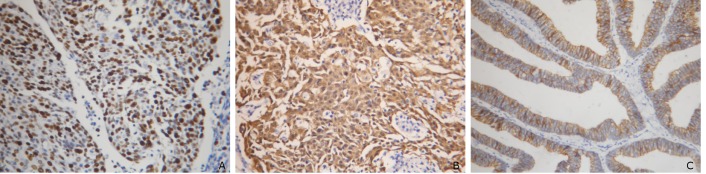
Immunohistochemical staining showed Bmi-1, P16, and CD44v6 protein expression in cervical mucous membrane. A, Bmi-1 protein; B, P16 protein; C, CD44v6 protein.

The expression of Bmi-1 protein mainly presented as the pale or dark-brown particles in the nucleus. A few light-brown particles were also observed in the cytoplasm (**Figure 1**). In the present study, the positive rate of Bmi-1 was 54.8% for the 62 UCC cases (34/62), among which the over-expression rate was 29.0% (18/62). The over-expression rates of Bmi-1 for the 30 cases with cervical neoplasia and the 20 cases with normal cervical mucosa were 3.3% (1/30) and 0%, respectively. Thus, the expression of Bmi-1 protein was significantly higher in the UCC group than in the adenoma and normal controls (*P*<0.05). No statistically significant difference was observed between the protein expression in the adenoma and normal control groups (*P*>0.05). The positive coloration was indicated by the light-brown particles, with coloration of the cell membrane in most cells, and that of the cytoplasm in a few cells (**Figure 1**). For the normal cervical mucosa, cervical neoplasia, and UCC tissues, the positive expression rates of P16 protein were 5.0% (1/20), 6.7% (2/30), and 61.3% (38/62), respectively. Statistical significant differences were observed among the three. The p16 protein expression (**Figure 1**) in UCC was separately compared with that in cervical neoplasia and the normal cervical mucous membrane, showing significant differences in both comparisons (*P*<0.01). However, there was no statistical difference between the protein expression in cervical neoplasia and normal cervical mucosa (*P*>0.05). Among the 62 UCC cases in the present study, 14 were over-expressed in the expression of CD44v6 protein (**Figure 1**), showing an over-expression rate of 22.6% (14/62). In the 30 cases with cervical neoplasia, over-expression was found in only one case (severe atypical hyperplasia), with an over-expression rate of 3.33% (1/30). From the 20 cases with normal cervical mucosa, all were under-expressed. Thus, the expression of CD44v6 protein was obviously higher in the UCC than in the adenoma and normal control groups (*P*<0.05). No statistical difference was observed between the expression in the adenoma and normal control groups (*P*>0.05).

### Relationship among the expression of Bmi-1, P16, and CD44v6 protein as well as the clinicopathologic features of UCC

The over-expression of Bmi-1 protein is both related to the distant metastasis of UCC and TNM staging, i.e., the expression of Bmi-1 protein was apparently higher in patients with distant metastasis than in those without distant metastasis (*P*<0.01), and Bmi-1 protein expression was higher in patients with stage-III/IV UCC than in those with stage-I/II UCC based on TNM staging (*P*<0.05). However, the over-expression was not correlated with the sex and age of the patients, tumor size, distribution, degree of differentiation, histological types, or status of metastasis (*P*>0.05) (**Table 1**). Logistic regression analysis further revealed that only the distant metastasis was related to the over-expression of Bmi-1 protein, i.e., distant metastasis was the most dangerous factor affecting the over-expression of Bmi-1 protein (*P*<0.01, B=1.705, OR=5.50) (**Table 2**). P16 protein expression was correlated with the sex and age of the patients, tumor size, distribution, degree of differentiation, histological types, nodal metastasis, and distant metastasis, as well as to clinicopathologic features such as TNM staging (*P*>0.05, **Table 1**). The over-expression of CD44v6 protein correlated to lymph node and distant metastasis, as well as the TNM classification (*P*<0.05). That is, the expression of CD44v6 protein was higher in patients with nodal and distant metastasis and in those with TNM stage-III/IV UCC, compared to the patients without nodal and distant metastasis, and those with TNM stage-I/II UCC. However, the over-expression of the protein had no relation with the age and sex of patients, size and distribution of tumor, degree of differentiation, or histological types (*P*>0.05). Logistic regression analysis further revealed that nodal metastasis was correlated with over-expression of CD44v6 protein (*P*<0.05, B=1.921, OR=0.146) (**Table 3**).

**Table 1:** Relationship of the expression level of Bmi-1, p16, and CD44V6 proteins with the clinicopathologic features of UCC.

Characteristics	*n*	Bmi-1 protein				p16 protein				CD44v6 protein		
		Under-expression	Over-expression	*P*		+	-	*P*		Over-expression	Under-expression	*P*
Age, years												
< 60	43	30	13	>0.0		23	20	>0.0		8	35	>0.05
≥ 60	19	13	6	5		15	4	5		6	13	
Tumor size, cm												
< 5	30	21	9	>0.0		18	12	>0.0		7	23	>0.05
≥ 5	32	22	10	5		20	12	5		7	25	
Differentiation												
Hi\Me	28	20	8	>0.0		17	11	>0.0		6	22	>0.05
Lo	34	23	11	5		21	13	5		8	26	
Histological types												
SqCa	58	40	18	>0.0		36	22	>0.0		13	45	>0.05
Adenoma	4	3	1	5		2	2	5		1	3	
Nodal metastasis												
Yes	23	6	17	<0.0		10	13	>0.0		13	10	<0.05
No	39	37	2	5		28	11	5		1	38	
Distant metastasis												
Yes	21	4	17	<0.0		12	9	>0.0		11	10	<0.05
No	41	39	2	5		26	15	5		3	38	
TNM Classification												
Stage-I/II	24	21	3	<0.0		11	13	>0.0		4	20	<0.05
Stage-III/IV	38	22	16	5		27	11	5		10	28	

**Table 2:** Multivariate logistic regression analysis of Bmi-1 protein expression.

Variables	B	Wald	*P*	OR	95%CI
Distant metastasis	1.809	7.536	0.003	5.80	2.11-17.23
Constant	-1.973	-	-	-	-

**Table 3:** Multivariate logistic regression analysis of CD44V6 protein expression.

Variables	B	Wald	*P*	OR	95%CI
Lymph node metastasis	0.924	3.672	0.008	2.7	0.92-9.32
Constant	-1.973	-	-	-	-

### Relationship of the expression of Bmi-1 and CD44v6 proteins with the prognosis of UCC patients

**Figure 2:**
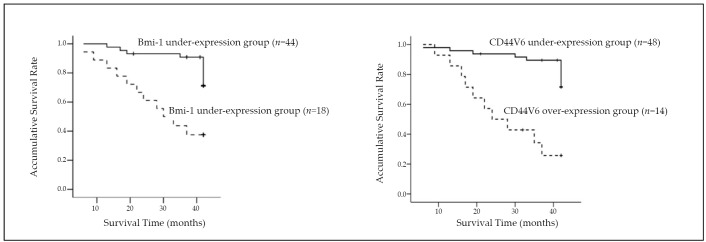
Kaplan–Meier survival curves of UCC patients with various expression levels of Bmi-1 and CD44V6 proteins.

A Kaplan–Meier survival curve was used to analyze the relationship between the Bmi-1 protein expression and the survival rate of the 62 UCC patients. The log-rank test was then used to analyse the statistical significance between the survival rates of the two groups. The results indicated that the survival rate was obviously lower in patients with over-expression of Bmi-1 protein than in those with under-expression (χ^2^=12.03, *P*=0.001). The survival rate was apparently lower in the patients with over-expression of CD44v6 protein than in those with under-expression (χ^2^=19.228, *P*=0.001) (**Figure 2**).

### Correlation between the expression of Bmi-1 and CD44v6 proteins in UCC

Spearman correlation analysis showed a positive correlation between the expression of Bmi-1 and CD44v6 proteins (*r*=0.419, *P*=0.001) (**Table 4**).

**Table 4:** Correlation between Bmi-1 and CD44v6 protein expression in UCC.

	CD44V6 (*n*)		Total	*r*	*P*
	High	Low			
Bmi-1 (high)	9	9	18	0.419	0.001
Bmi-1 (low)	5	29	34		

## Discussion

UCC is one of the most common malignancies in gynecological oncology. Over the past few years, the incidence rate of UCC has increased, and the affected population has become younger. The oncogenesis and progression of UCC is a complicated course involving multiple steps and genes^[^^11^^]^. The human Bmi-1 gene is located at zone 13 of the 10th short arm of the chromosome, i.e., 10p13, which contains 10 exons and 10 introns. The NK4a/ARF site is the downstream control site of the Bmi-1 gene, which is negatively regulated immediately by the Bmi-1 gene so as to affect the proliferation and aging of the cells ^[^^2^^]^. Studies over the past few years have proven that Bmi-1 directly participates in the oncogenesis, progression, invasion, and metastasis of breast cancer ^[^^6^^]^, head and neck tumor ^[^^7^^]^, as well as gastric cancer ^[^^8^^]^. Bmi-1 may be a predictor of unfavorable prognosis of patients with these cancers.

The findings of the present study demonstrated that Bmi-1 protein expression had a tendency of up-regulation in normal cervical mucosa, cervical carcinoma, and UCC. The over-expression of the Bmi-1 protein in UCC suggested the relation of this protein to the oncogenesis and progression of UCC. This study found significant differences in the expression of p16 protein among UCC, cervical neoplasia, and normal cervical mucosa (*P*<0.01). The p16 protein expression was obviously higher in UCC than in the cervical neoplasia and normal cervical mucosa (*P*<0.01). No statistical difference was found in p16 protein between cervical neoplasia and normal cervical mucosa (*P*>0.05). Therefore, Bmi-1 and p16 play key roles in the oncogenesis of UCC. Oncogenesis has been suggested as the result of a dynamic disequilibrium between the proliferation and apoptosis of cells. Bmi-1 protein negatively regulates the INK4a/ARF site, thus lowering the expression of p16INK4a and p19ARF proteins, increasing cell proliferation, and decreasing apoptosis ^[^^2^^]^. The p16 gene is the first anti-oncogene found in recent years that directly partakes in the regulation of the cell cycle, which is closely related to the oncogenesis and progression of several tumors. The p16 gene directly regulates the G- and S phases of the cell cycle by the coded p16 protein, and negatively regulates the growth and proliferation of cells. Abnormalities in the p16 gene, such as deletion, point mutation, and methylation, may result in decreased p16 protein expression and a runaway cell proliferation, causing tumorigenesis ^[^^12^^]^. In the present study, both Bmi-1 and p16 have been observed to be over-expressed in UCC. These two proteins may jointly regulate the cell cycle, and may result in an abnormal proliferation of cells, causing the progression of the epithelial cells of normal cervical mucosa toward UCC.

Invasion and metastasis are the major features of cancer cells. In this study, the over-expression rate of Bmi-1 protein was not related to the sex and age of the patients, tumor size, distribution, degree of differentiation, histological types, or possibility of metastasis. Nevertheless, Bmi-1 protein was related to distant metastasis and TNM staging. Further results of the logistic regression analysis indicated that only the distant metastasis was registered in the model, demonstrating that distant metastasis was the most correlated factor for the over-expression of Bmi-1 protein. The results suggested that the over-expression of Bmi-1 protein in UCC was one of the pridictive factors for invasion and metastasis of UCC. However, the mechanism of Bmi-1 in regulating the invasion and metastasis of UCC remains unclear. Previous studies have shown that for cases with several malignant tumors, the rate of recurrence and nodal metastasis is quite high in patients with positive CD44v6 expression^[^^10^^]^. CD44v6 gene expression is also related to the invasion and metastasis of cancer. Therefore, the current study tentatively concludes a possible correlation between the expression of Bmi-1 protein and CD44v6 protein. This study found that a higher expression of Bmi-1 protein indicated a higher expression of CD44v6 protein. Spearman correlation analysis further revealed a positive correlation between the expression of Bmi-1 and CD44v6 proteins in UCC. Concurrently, an analysis of the Kaplan-Meier survival curve indicated that the survival rate was obviously lower in patients with Bmi-1 or CD44v6 over-expression than in those with under-expression. Therefore, Bmi-1 may play a part in the regulation of CD44v6, but the mechanism needs further investigations.

Oncogenesis and tumor progression involve many steps and genes. The current study has proven that Bmi-1, P16, and CD44v6 jointly participate in the oncogenesis and progression of UCC. The abnormal expression of Bmi-1 and p16 brings about the disorder and the abnormal proliferation of normal cervical epithelial cells, resulting in UCC. Successive over-expression of Bmi-1 may activate the abnormal expression of CD44v6, causing the invasion and metastasis of UCC.
